# Cell Death by *Toxoplasma gondii*

**DOI:** 10.1590/0037-8682-0530-2019

**Published:** 2020-06-12

**Authors:** Claudio Bruno Silva de Oliveira, Isabelle Luna de Oliveira Dantas Berto, Valter Ferreira de Andrade

**Affiliations:** 1 Universidade Federal do Rio Grande do Norte, Departamento de Microbiologia e Parasitologia, Laboratório de Biologia da Malária e Toxoplasmose, Natal, RN, Brasil.; 2 Faculdade UNINASSAU, Curso de Biomedicina, Natal, RN, Brasil.

**FIGURE 1:**
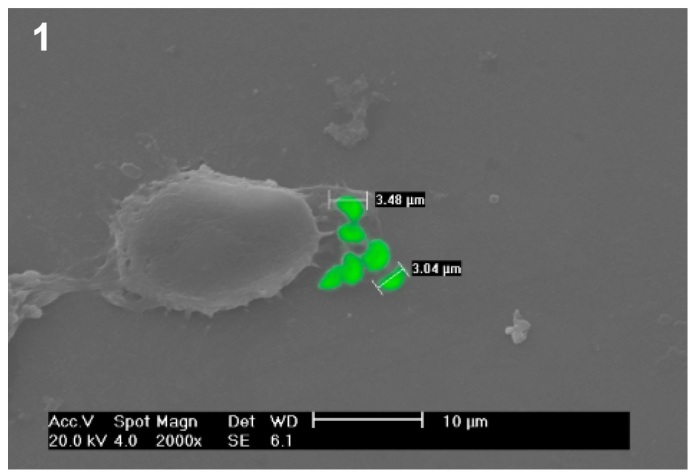
*Toxoplasma gondii* tachyzoites around a Hela cell after the cell culture is infected. Scanning electron microscopic image. **FIGURE 2:** *Toxoplasma gondii* tachyzoites after death of the infected Hela cell, approximately 20 h after infection. Scanning electron microscopic image.

**FIGURE 2:**
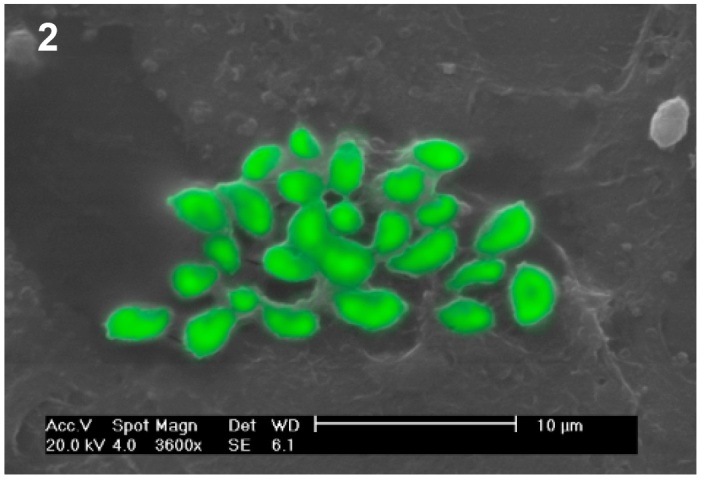
*Toxoplasma gondii* tachyzoites after death of the infected Hela cell, approximately 20 h after infection. Scanning electron microscopic image.

*Toxoplasma gondii* is a dangerous intracellular parasite that can cause toxoplasmosis, a disease that has several clinical manifestations^1^. One explanation for its diverse clinical features is the wide variability in parasite strains across the globe^2^. Here, we used scanning electron microscopy to reveal the instant when a group of parasites kills a cell. These parasites can infect any nucleated cell^3^ and a similar mechanism occurs in human cells. In Figure 1, *T. gondii* tachyzoites are observed immediately after infection in the HeLa cell culture. In the image, the small size of the parasites can be compared to the cell that will be parasitized. In Figure 2, approximately 20 h after infection, the dead cell can be seen owing to the remarkable reproductive capacity and pathogenicity of this parasite.
